# Oral Pathogenic Bacteria and the Oral–Gut–Liver Axis: A New Understanding of Chronic Liver Diseases

**DOI:** 10.3390/diagnostics13213324

**Published:** 2023-10-26

**Authors:** Yumeng Lei, Simin Li, Mingxin He, Zichun Ao, Jiadun Wang, Qingming Wu, Qiang Wang

**Affiliations:** Hubei Province Key Laboratory of Occupational Hazard Identification and Control, Institute of Infection, Immunology and Tumor Microenvironment, School of Medicine, Wuhan University of Science and Technology, Wuhan 430065, China; lym8252022@163.com (Y.L.); lsm3667@163.com (S.L.); mingxin_he2021@163.com (M.H.); 18871226539@163.com (Z.A.); wangjiadun@wust.edu.cn (J.W.); wuhe9224@sina.com (Q.W.)

**Keywords:** oral–gut–liver axis, oral pathogenic bacteria, oral microbiota, chronic liver diseases, hepatocellular carcinoma

## Abstract

Liver diseases have long been a prevalent cause of morbidity and mortality, and their development and progression involve multiple vital organs throughout the body. Recent studies on the oral–gut–liver axis have revealed that the oral microbiota is associated with the pathophysiology of chronic liver diseases. Since interventions aimed at regulating oral biological disorders may delay the progress of liver disease, it is crucial to better comprehend this process. Oral bacteria with potential pathogenicity have been extensively studied and are closely related to several types of chronic liver diseases. Therefore, this review will systemically describe the emerging role of oral pathogenic bacteria in common liver diseases, including alcoholic liver disease (ALD), non-alcoholic steatohepatitis (NASH), non-alcoholic fatty liver disease (NAFLD), cirrhosis, autoimmune liver diseases (AILD), and liver cancer, and bring in new perspectives for future research.

## 1. Introduction

Despite the great efforts made in the last decade to fight liver disease, it still has a major impact on global health. A recent report updated the global burden of liver disease: 2 million deaths worldwide (1 in 25 deaths globally), one in three female liver deaths, the 11th leading cause of death globally, and the 15th leading cause of disability-associated life years [1]. In 2016, liver disease-related expenditures in the United States were USD 32.5 billion, with healthcare expenditures increasing 4% annually for more than 20 years [2]. It is estimated that more than one-fifth of the Chinese population is affected by some form of chronic liver disease (CLD), particularly cirrhosis, hepatocellular carcinoma (HCC), NAFLD, and ALD. CLD and its complications are responsible for significant morbidity and mortality worldwide [3].

The gut microbiota and numerous liver disorders are closely related [4]. Recent evidence suggests a potential link between oral microbial dysbiosis and the occurrence and development of liver disorders; however, the precise influencing mechanism is still unclear [5]. Therefore, in order to develop more effective strategies for preventing and treating liver diseases, it is crucial to comprehend the invasion route of oral pathogenic bacteria as well as any potential biological aspects. The intestine and the oral cavity are part of the same gastrointestinal tract and are related anatomically. There is increasing evidence that oral microbiota can reach the gastrointestinal tract through the bloodstream or intestinal route [6]. The transfer of oral microbiota to the intestinal tract has the potential to aggravate various intestinal conditions and, more importantly, may contribute to the development of liver disease by affecting the intestinal microbiota [7]. This review article aims to highlight the role of oral pathogenic bacteria such as *Porphyromonas gingivalis* (*P. gingivalis*), *P. intermedia*, and *S. pyogenes* in various liver diseases and to explore the oral–gut–liver axis as a new perspective and direction for future research.

## 2. Oral Microecology

According to information from the Human Oral Microbiome Database (HOMD), there are 774 species of oral bacteria in the oral cavity. The homeostasis of the oral microbiota is of great importance for oral diseases and systemic diseases, especially liver diseases. Dental caries (tooth decay), periodontitis, gingivitis, and pulp abscesses are only a few of the oral illnesses that can arise from dysbiosis of the oral microbiota [8].

Periodontitis is a chronic inflammatory disease caused by bacteria in the oral cavity. As inflammation persists, periodontal tissues such as gums, periodontal ligaments, cementum, and alveolar bone are destroyed. The periodontal bacteria detected in the subgingival pocket of patients with periodontitis and related to the occurrence and development of periodontitis include *P. gingivalis*, *Tannerella*, *Treponema pulposus*, *Proctella intermedia*, and *Actinobacteria* [9].

*P. gingivalis* is present in healthy populations and is recognized as a host-adapted pathogen that can cause pathogenicity only when the ecological balance between host and bacteria is disrupted. Also, the bacterium may be involved in regulating the development of plaque biofilms, coordinating and controlling their pathophysiological mechanisms to minimize damage to the host. In particular, during the inflammatory process of chronic periodontitis, *P. gingivalis* has the ability to evade the host’s immune response and obtain access to nutrients in the microenvironment, and its survival and proliferation are directly related to the development of periodontitis [10].

In recent years, the primary virulence factors utilized by *P. gingivalis* have been identified and characterized, including a mixture of toxins, mainly proteases known as gingival proteases, which promote the invasion of gingival tissues [11]. The main virulence factor of *P. intermedia* 17 (Pi17) is mainly the extracellular mucopolysaccharide (EPS) [12]. *A. actinomycetemcomitans* is a Gram-negative pathogenic anaerobic bacterium that causes periodontal diseases such as limited invasive periodontitis (LAP) and periodontitis. Leukotoxins, lipopolysaccharides (LPSs), surface-associated substances, and enzymes are the principal pathogenic factors of common actinomycetes [13]. In addition, *S. mutans* is a major caries-causing bacterium with a variety of caries-related characteristics, including EPS synthesis, biofilm formation, acid production, and acidity [14], which is also proposed to be a cause of non-alcoholic fatty liver disease [15]. *P. intermedia* readily colonizes healthy dental biofilms and has been associated with periodontal disease [16]. The names of certain oral pathogenic species and their properties are listed in Table 1.

When the oral microecology is disturbed, various pathogenic bacteria, as previously mentioned, will produce virulence factors, which can cause diseases not only in the mouth but also in other areas of the body, such as the intestine and liver [17]. Apart from oral health issues, a large amount of evidence indicates that oral bacteria are closely related to systemic diseases as well, including, but not limited to, inflammatory bowel diseases (IBD) [18], diabetes [19], brain health [20], liver diseases [21], and other conditions. Furthermore, the oral microbiota has also been associated with various kinds of cancer development [8]. Nevertheless, there is a bi-directional relationship, and the oral microbiome is simultaneously modified by systemic diseases [22,23]. Collectively, the oral microbial ecosystem is of great significance to human well-being.

## 3. Oral–Gut–Liver Axis

The oral cavity and the gut are the two major microbial reservoirs in the human body [24]. However, some differences exist in the distribution of the oral and intestinal microbiota because of the presence of the oral–intestinal barrier [25]. However, there are certain differences in the distribution of oral and intestinal microbiota because of the existence of the oral–intestinal barrier [26]. On the other hand, in the case of oral–gut barrier dysfunction, oral microbiota can be transferred to the intestinal mucosa more easily [27]. For instance, studies have revealed that typical pathogenic bacteria in the mouth (*F. nucleatucm* and *P. gingivalis*) can be found in the colon of periodontitis patients [28]. There is also increasing evidence linking the oral microbiota to the occurrence and development of gastrointestinal cancer [29]. Therefore, scholars proposed the view of the “oral–gut–axis”. Ectopic colonization of oral microbiota, such as *P. gingivalis*, *S. mutans*, and *F. nucleatum*, may lead to the destruction of the intestinal epithelial barrier, excessive secretion of inflammatory factors, destruction of the host immune system, and imbalance of the intestinal microbiota, thus aggravating chronic intestinal inflammation and then triggering or aggravating intestinal diseases [30].

Marshall first put forward the concept of the “gut–liver axis” in 1998 [31]. The gut-liver axis consists of two-way interaction/communication pathways between the gastrointestinal tract and the liver. Intestinal-derived products can be transported to the liver via the biliary tract, portal vein, and systemic circulation, affecting various liver functions. Moreover, intestinal-derived products can also be transported to the intestines via hepatic feedback pathways, controlling intestinal metabolic functions and affecting the integrity of the intestinal barrier and the composition of the microbiota [32]. On the one hand, the liver can directly affect the reproduction of the intestinal gut microbiota by secreting bile acid, regulating the absorption of intestinal substances and the proliferation of colon cells, and affecting the intestinal mucosal barrier function and intestinal environment [33]. On the other hand, the increase in intestinal microbial transfer to the liver may lead to more severe liver damage [34]. Intestinal microbiome imbalance will lead to increased levels of LPSs in the gut, the liver translocation of LPSs under the action of portal vein circulation, and the high expression of Toll-like receptor 4 (TLR-4) in the liver, while TLR4 mediated TGF-β. The signal will result in liver inflammation, fibrosis, and HCC if it is continuously activated [35].

**Figure 1:**
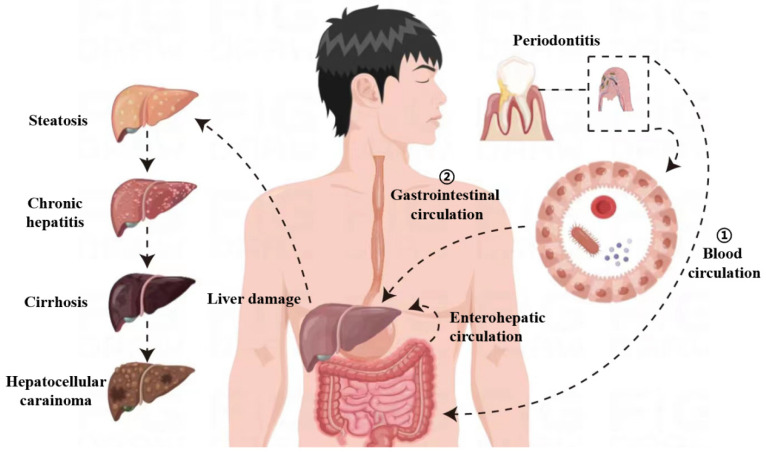
Oral pathogenic bacteria can disseminate by different routes—haematogenous or gastrointestinal—to reach the liver, where they can cause or exacerbate inflammatory pathologies. When periodontitis occurs, oral pathogenic bacteria can enter the blood circulation through the damaged oral mucosa, thereby reaching the liver. Alternatively, oral pathogenic bacteria may enter the gastrointestinal circulation by swallowing saliva, causing intestinal biological disorders and intestinal barrier function damage, thus contributing to endotoxemia and inflammation. Then, due to the presence of enterohepatic circulation, these effects may directly trigger or exacerbate pre-existing liver damage through detrimental metabolic consequences, resulting in liver steatosis, chronic hepatitis, fibrosis, and cirrhosis, and the progression of cirrhosis to hepatocellular carcinoma.

With the introduction of the “oraltgut axis” and “guttliver axis”, people gradually discovered that the oral microbiota can communicate with the liver through the gastrointestinal tract, blood, and portal circulation. Oral bacteria and their metabolites, after arriving at the gut and being absorbed there, are directly transmitted to the liver through the portal vein or employed as energy or as signal and regulatory molecules to participate in liver metabolism [36]. According to an in vitro study, swallowing periodontal pathogenic bacteria can exacerbate NAFLD, possibly by inducing intestinal ecological dysregulation and a subsequent influx of intestinal bacteria and/or bacterial products leading to dysregulated gene expression [37]. The diagram of the oral–gut–liver axis is shown in Figure 1.

The liver is the organ in closest contact with the gut, where the oral microbiota is prevalent, and is exposed to a high variety of bacterial components and metabolites [38]. There has been evidence of possible crosstalk between the oral microbiota, gut microbiota, and liver function in liver disease. Studies in both human and animal models have indicated that colonic inflammation increases intestinal permeability in patients with liver disease, leading to the translocation of bacteria and bacterial products to the liver, in which case bacteria from the oral cavity or their by-products have the potential to disseminate to the liver, thereby contributing to the pathogenesis of liver disease [39,40,41]. Significant changes in the gut microbiota of cirrhotic patients may be related to the invasion of the gut by oral bacterial species, and genetic tracing has shown that most of them are of oral origin [42]. The altered collateral phenomena made possible by the reduced bile and gastric acid secretion present in cirrhosis have also been theorized to render the intestinal tract more easily admissible or accessible to oral pathogens [43]. In summary, oral bacteria can be transferred to the gastrointestinal tract, where they contribute to inflammation and tumorigenesis, and oral bacteria colonizing the intestinal tract can further disseminate to the liver and cause liver lesions.

## 4. Liver Diseases and Oral Pathogenic Bacteria

Changes in the oral microbiota related to liver diseases are shown in Table 2.

### 4.1. Alcoholic Liver Disease and Oral Pathogenic Bacteria

Every year, ALD claims the lives of more than 3.3 million people worldwide [49]. Compared to non-Asian populations, some Asian populations have a higher risk of developing cirrhosis and cancer due to excessive alcohol consumption [50]. The disruption of the microbiota is an important risk factor for ALD, in addition to alcohol consumption. A significant increase in pathogenic bacteria and toxic metabolites in the oral cavity can transfer to the liver through the gastrointestinal tract or blood circulation, leading to liver damage [32].

A recent study has pointed out that there is a potential correlation between oral bacterial dysbiosis and ALD [45]. There is a general reduction in bacterial diversity in the microbiome of patients with all forms of ALD [51]. Fan et al. found that drinkers might own a varying oral bacteria community composition compared to non-drinkers, including increased colonization of potentially pathogenic bacteria [52]. More profoundly, Zhou et al. provide preliminary data, supporting the notion that *P. gingivalis* may be related to ALD and function as a novel risk factor in both the onset and severity of acute alcoholic hepatitis (AAH) [53]. An elevated antibody response to *P. gingivalis* was observed in AAH in this study. Plasma anti-*P. gingivalis* IgM, IgG, and IgA were significantly elevated in patients with severe AAH, revealing that *P. gingivalis* may be a novel risk factor for the progression and severity of AAH. Consequently, targeting oral pathogens seems to provide an underlying approach to the prevention and treatment of ALD [54].

### 4.2. Non-Alcoholic Fatty Liver Disease and Oral Pathogenic Bacteria

To date, there have been a number of exciting findings regarding the oral microbiota and its role in NAFLD progression [47,55]. Periodontitis can worsen a simple fatty liver into a fibrotic disease through the production of fibrosis mediators such as transforming growth factor-β1 (TGF-β1) and galectin-3 [56]. The incidence of oral pathogenic bacteria was compared between patients with NAFLD and control subjects, and it was found that patients with NAFLD had a significantly higher detection rate of *P. gingivalis* infection [57]. In addition, the tooth infection caused by *P. gingivalis* might worsen the development of a NASH liver from a simple fat liver to a fat liver and make it worse and worse [44]. However, the elimination of *P. gingivalis* can inhibit liver fibrosis and inflammation in NASH [58].

On the one hand, the colonization of *P. gingivalis* in the liver found in pathological sections may be an important cause of liver injury. Atsuhiro et al. demonstrated that odontogenic infection with *P. gingivalis* exacerbates fibrosis in NASH via hepatic stellate cell activation [47]. *P. gingivalis* induces apoptosis by upregulating phosphor-smad2 (TGF-β 1’s key signaling molecule) and immunoexpression of galectin-3, which enables macrophage accumulation and a dramatic increase in the number of hepatic crown structures associated with liver fibrosis, as well as the area of fibrosis. Yao et al. showed that *P. gingivalis* may also induce the development of a nonalcoholic fatty liver through iron death [59]. On the other hand, one possible mechanism is that the bacteria *P. gingivalis* can transport protease to the liver through the outer membrane vesicles and then affect the liver cells. For example, Mariko et al. showed that the outer membrane vesicles (OMVs) of *P. gingivalis* could attenuate insulin-induced Akt/glycogen synthase kinase-3 β signaling in a vinculin-dependent manner in HepG2 cells [60].

Currently, targeted drug therapy for NAFLD is limited, and liver transplantation is considered the optimal treatment [61]. However, the prognosis after liver transplantation is poor in terms of survival and high tumor recurrence rates, and the development of novel therapies is urgently needed. Bajaj et al. found that periodontal treatment was associated with the improvement of oral microbial dysbiosis and systemic inflammation by assessing endotoxemia and inflammation perception [62]. Notably, there seems to be an association between periodontitis and serum ALT levels. In a Japanese study, it was found that men with high serum ALT levels were more likely to develop periodontitis than those with low serum ALT levels [57]. Yoneda et al. performed periodontal treatment, including oral scaling and root planing, topical administration of minocycline hydrochloride, and hygiene instruction, in patients with NAFLD suffering from periodontitis [57]. The results showed that AST and ALT levels decreased 1 month after baseline, and the decline reached statistical significance after 2 months; after 3 months, a further decrease was observed [57]. These advances have enriched our understanding of the mechanisms of the “oral–gut–liver axis” and have confirmed the positive effects of periodontal therapy on the management of liver disease.

### 4.3. Cirrhosis and Oral Pathogenic Bacteria

Quantitative metagenomics revealed that most gut-enriched, taxonomically assigned species in cirrhotic patients originated from the oral cavity and differed significantly at the gene and functional levels [42]. A cohort study by Fernando et al. observed an important risk association between cirrhosis and periodontitis, with a higher prevalence of periodontitis in patients with cirrhosis compared to healthy controls [63]. Lea et al. achieved similar results, demonstrating a substantial correlation between periodontitis and mortality, complications, and nutritional risk scores in patients with cirrhosis [64]. Therefore, some scholars believe that periodontitis is a risk factor for infection in patients with liver cirrhosis, and periodontal treatment for patients with liver cirrhosis may have a mitigating effect [65].

Anders et al. used long-read Illumina sequencing to analyze the subgingival microbiome of 21 patients with periodontitis and liver cirrhosis [48]. The subgingival microbiota is mainly composed of bacteria belonging to the *Firmicutes* phylum, with *Actinomycetes* and *Bacteroidetes* phyla in a smaller range [48]. It is well known that patients with periodontitis have high serum levels of antibodies to *P. gingivalis*. There have been clinical reports of a morbidly obese patient with NASH-related cirrhosis who died of sepsis caused by a *P. gingivalis* infection [66]. This case may represent an important implication of *P. gingivalis* in the progression of cirrhosis in patients with NASH.

It has been proven that systemic periodontal therapy in patients with liver cirrhosis can improve endotoxemia, systemic and local inflammation, and the abnormal microbial metabolism in saliva and feces within 30 days. However, in the cirrhosis group without periodontal treatment, endotoxin and LPS-binding protein were increased for identical durations [62]. Therefore, periodontal therapy may be a potential adjunctive therapy to prolong survival in patients with cirrhosis.

### 4.4. Hepatocellular Carcinoma and Oral Pathogenic Bacteria

Hepatocellular cancer (HCC) is about the sixth most common cancer form, accounting for approximately 9.2% of all cancer-related mortality [27]. Most patients with HCC have developed chronic liver disease, which is caused by NAFLD and alcohol-related fatty liver disease.

Comprehensive analysis of the microbiome and host transcriptome has illustrated the association between the gut microbiota and clinical outcomes in HBV-associated HCC. The change in oral microorganisms may also accelerate the occurrence of HCC. Brende et al. provided evidence that the oral microbiome of HCC cases showed significantly lower alpha diversity and significant differences in the relative abundance of 30 taxa compared to healthy controls, with *Cyanobacteria* being enriched in HCC [67]. Microcystins produced by cyanobacteria are the most widely distributed hepatotoxins and are strong promoters of liver tumors. Therefore, oral *Cyanobacteria* may, directly and indirectly, promote liver tumors through microcystins and other hepatotoxins, serving as an independent risk factor for HCC. Li et al. detected the diversity of oral bacteria in patients with different liver diseases and healthy controls by 16SrDNA high-throughput sequencing and bioinformatics analysis [31]. It was discovered that *P. gingivalis*, *Epsilon proteobacteria*, *Actinobacteria*, *Clostridia*, and *Fusobacteria* had a higher abundance in patients with HCC, and with the development of liver diseases, the species of saliva bacteria decreased. This might provide useful information and possible biological symbols for patients with liver disease. Furthermore, an epidemiological study has shown a positive association between periodontal diseases and cancers (including HCC) [68]. Yang et al. examined the association between tooth loss and the incidence of primary HCC in a prospective cohort of Finnish male smokers and found that more tooth loss was associated with a higher risk of primary HCC [69].

Robert et al. revealed the mechanism of the intestinal hepatic axis promoting the development of HCC, including biological disorders, intestinal leakage, and bacterial metabolites [70]. LPSs released by periodontal bacteria combine with TLR-4 and TLR-2 to stimulate T lymphocytes and macrophages to produce cytokines and proinflammatory mediators [71]. The production of these factors may induce an increase in NADPH oxidase 4 (NOX-4) and malondialdehyde levels in the liver and a decrease in glutathione, catalase, and selenium levels, which would lead to the decline of liver function, continuous inflammation of the liver, and the accelerated occurrence of HCC. Furthermore, as the first pathogenic bacteria of periodontitis, *P. gingivalis* can change the fatty acid profile in the tongue tissue and serum of mice, thereby promoting the development of cancer [72]. Interestingly, studies have shown that HGFs are involved in the development of oral cancer, which indirectly demonstrates a significant association between oral disease and liver disease [73]. The human microbiome is valuable for the diagnosis of HCC and provides a new strategy for the targeted treatment of HCC [74]. Oral microbiology has great potential for clinical application and may be widely used in the future for the diagnosis, treatment, and prognosis of liver diseases and cancers, bringing additional benefits to the HCC population, and the removal of periodontal pathogens may be a new direction in the diagnosis and treatment of HCC.

### 4.5. Autoimmune Liver Disease and Oral Pathogenic Bacteria

The oral microbiome is a key regulator of immunity based on the association between dysregulated oral microecology and autoimmune disease, loss of immune tolerance to autoantigens, and increased inflammatory events [75]. Epidemiological evidence has suggested that bacterial infection is associated with primary biliary cholangitis (PBC), and bacterial infection is considered to be one of the most important environmental factors contributing to the disruption of mitochondrial autoantigen tolerance in PBC [76]. A series of studies also revealed that disruption of tolerance to mitochondrial autoantigens through *E. coli* infection may result in auto-reactive T lymphocytes and B lymphocytes recognizing autoantigens [77]. *E. coli* is not the only candidate for breaking tolerance to mitochondrial autoantigens; other bacteria, such as *Novosphingobium aromaticivorans* and *Lactobacillus delbrueckii*, may be involved in the etiology of PBC through molecular mimicry [76].

Although the initiating factors of autoimmune hepatitis (AIH) etiology are unknown, the role of the oral–gut–liver axis in the pathogenesis of AIH has attracted attention. Rao et al. collected 204 saliva samples (from 68 AIH patients and 136 healthy controls) and performed microbial sequencing and found increased oral microbial diversity in the AIH and differences with the overall oral microbial composition of healthy controls both at the phylum and genus levels [78]. Another study evaluated the correlation between immune biomarkers measured by Bio-Rad and the oral microbiome of patients with PBC and AIH, both of which differed from healthy controls in oral microbiota composition and abundance [46]. Immunologic biomarker analysis revealed elevated levels of inflammatory cytokines and immunoglobulin A in the saliva of patients with autoimmune liver diseases (AILD) and a positive correlation between the relative abundance of *Veillonella* and the levels of IL-1β, IL-8, and immunoglobulin A in saliva, as well as the relative abundance of *Lactobacillales* in stool. In addition, Rao et al. established that several gene functions of the AIH group were proven to be advantageous, including membrane transport, transporter, and flagellum assembly. The main virulence factors of certain oral pathogenic bacteria, such as *P. gingivalis* and *F. nucleatum*, are known to be proteases, bacterial hairs, iron uptake transport proteins, and toxic outer membrane vesicles, leading to a local inflammatory response [79].

These findings suggest that the oral microbiota and associated inflammatory factors may serve as potential biomarkers to aid in the diagnosis of the onset and progression of AIH, but the specific mechanisms of the modulatory effects of the oral microbiota on AIH remain to be further elucidated.

## 5. Prevention and Management

### 5.1. Oral Hygiene

Patients who receive good oral care through improved hygiene practices experience a shift in the composition of their oral microbiota toward a healthy state, with a reduction in the total number of bacteria in saliva and plaque and a reduction in the proportion of periodontal pathogens. Since the microbial load in the oral cavity correlates with the severity of periodontal disease, oral hygiene is crucial for controlling the bacterial load in the oral cavity to help avoid inflammation and bacteremia, consequently maintaining oral health and lowering the risk of liver disease [80,81]. Oral hygiene measures proven to be beneficial include brushing with fluoride toothpaste and antibacterial mouthwash to reduce plaque, which can be effective in relieving gum inflammation [82,83]. There may be a negative correlation between the frequency of brushing and the prevalence of NAFLD, as reported in a cross-sectional study [84]. Several intervention studies have demonstrated that oral hygiene instruction and scaling/root planing (SRP) significantly improve AST and ALT levels, endotoxin levels, and liver fat content (LFC) in patients with liver disease, with a concomitant significant reduction in Porphyromonas gingivalis immunoglobulin G (IgG) antibody titers [56,57,85].

The aforementioned studies support the potential impact of oral hygiene on improving liver function and also reinforce the causal relationship between periodontal disease and NAFLD. However, more evidence is needed in the future to clarify the effectiveness of oral hygiene care in the treatment of liver disease.

### 5.2. Antimicrobial Peptides

Antimicrobial peptides are important contributors to maintaining balance in the oral environment and regulating oral biofilm microecological dysregulation through broad-spectrum antimicrobial activity. Through broad-spectrum antimicrobial activity, they can kill pathogenic microorganisms, promote tissue healing, and play a significant role in several oral diseases (e.g., caries, periodontal disease, mucosal disease, etc.) [86]. Epithelial cells produce salivary antimicrobial peptides, such as β-defensins, α-defensins, and LL-37, which not only act as bactericidal substances, but also as alarm signals when pathogens invade [87]. Notably, LL-37 has antifungal, antibacterial, and anti-biofilm properties, conditioning symbiotic actinomycetes, inhibiting the formation of biofilm by this bacterium, and preventing the growth of *F. nucleatum* [88]. The effect of LL-37 on Gram-negative bacteria may be particularly important, especially some harmful bacteria associated with rapidly progressive periodontal disease [89].

Several findings have highlighted the pathophysiological role of antimicrobial peptides in the progression of liver disease: Antimicrobial peptides inhibit intestinal inflammation-promoted LPS metastasis, liver inflammation, and fibrogenesis in NASH [90]; Cathelicidin-related antimicrobial peptides (CRAMP) modulate inflammatory vesicle activation by reducing LPS binding and UA production, thereby alleviating alcoholic liver disease [69]; In a mouse model of alcoholic hepatitis (AH), 1Z1 treatment upregulated the expression of the antimicrobial peptides Reg3b and Reg3g in the intestinal epithelium, reducing and increasing the number of *Bacteroides* and *Lactobacillus*, respectively, to regulate the microbiome and prevent intestinal barrier disruption and bacterial translocation, thereby inhibiting ethanol-induced liver injury, steatosis, and inflammation [91]. LL-37 has positive effects on different liver diseases: it is involved in regulating cell proliferation and the production of pro-inflammatory cytokines in hepatocellular carcinoma cells, and it also reduces hepatitis C virus infection [92]. Therefore, moderate supplementation with antimicrobial peptides may have a positive effect on regulating the oral microbiota and thus alleviating liver disease.

### 5.3. Prebiotics and Probiotics

Scientific evidence for probiotics and prebiotics confirms that they are microbiota management tools that improve host health [93]. In recent years, prebiotics and probiotics have been applied to the oral microbiome [94]. Administration of some probiotics and prebiotics can alleviate metabolic liver diseases such as NAFLD and NASH, which may progress to cirrhosis and hepatocellular carcinoma [95]. Prebiotic intervention reduces liver weight and steatosis, as well as hepatic adipogenic gene expression, hepatic inflammation, endotoxemia, and hepatic TLR4 signaling [96]. Probiotics manage periodontal disease by assisting in reducing oral damage from harmful bacteria, regulating local and systemic immune responses, and reducing plaque and gum inflammation [97]. Research data showed that *Lactobacillus* has antibacterial activity against oral pathogenic bacteria such as *S. mutans*, *P. gingivalis*, *P. intermedia*, and *A. actinomycetemcomitans*. The positive effect of probiotics and prebiotics on various liver diseases (hepatic encephalopathy, cirrhosis, and NAFLD) has been demonstrated [98]. Administration of *Bifidobacterium* and *Lactobacillus* has been shown to improve liver transaminase levels and histological lesions [99]. In conclusion, prebiotics and probiotics not only maintain oral health homeostasis by regulating the oral microbiota, but also serve as effective adjuvants to the conventional treatment of liver disease without side effects on the host.

## 6. Discussion

There is growing evidence that periodontitis is a risk element for the development of numerous systemic diseases, including liver disease. The interplay of immunological factors, genetic vulnerability, intestinal microorganisms, and environmental variables—of which compromised immune regulation plays a crucial role—determines the etiology and pathophysiology of liver illnesses. The incidence rate of periodontitis, the depth of the periodontal pocket, and the rate of tooth loss in patients with liver disease are significantly higher than in healthy controls, which are closely linked to changes in the oral microbiota [100]. In addition, periodontitis-related oral bacteria might transfer to the gut and, along with the intestinal microbiota, contribute to the barrier dysfunction of intestinal epithelial cells, thus aggravating liver damage.

To elucidate the role of oral pathogenic bacteria in diseases, we summarized several mechanisms of their remote transmission and liver damage. Due to the interconnectivity between the oral cavity and the intestine, a large number of oral bacteria can continuously enter the intestine through saliva, causing disruption of the intestinal microbiota, increasing intestinal epithelial permeability, or causing endotoxemia [101]. In addition, oral pathogens, such as *P. gingivalis*, can output a variety of virulence factors [60] and reduce innate immunity and adaptive immunity, which enables pathogens to survive and reproduce in the host, thus destroying periodontal tissue and participating in the occurrence and development of systemic diseases [102]. The recombination of antimicrobial peptides and the destruction of phagocyte function contribute to the escape of *P. gingivalis*.

Biomedical research in the past decades has produced a large amount of evidence proving that *P. gingivalis* has contributed to the development of various liver diseases [103]. In this review, we summarized the existing evidence of the association between *P. gingivalis* as a risk regulator and different liver diseases from the perspective of epidemiological and biological research experiments. According to recent research results, we believe that *P. gingivalis* may be a key susceptibility factor in the microenvironment, which guides the development or poor prognosis of different liver diseases and cancers [55,104]. Several studies have reported the relationship between specific antibodies to *P. gingivalis* in serum and liver diseases [105]. In saliva specimens from patients with cirrhosis or previous rounds of hepatocellular carcinoma treatment, 50% contained FimA protein from *P. gingivalis*. The formation of purulent liver abscess (PLA) has three main routes of infection: hepatic artery, biliary tract, and portal vein; nevertheless, 50% are of implicit origin (source unknown). A case report showed that periodontal infection was a latent cause of PLA formation. Gingival bacilli and other periodontal pathogens isolated from the PLA, heart, kidney, and thrombus of the patient were detected by immunohistochemical staining [106]. Studies have shown that LPSs derived from *P. gingivalis* can cause excessive accumulation of liver lipids by activating NF-κB and JNK signaling pathways, thus leading to the occurrence and development of liver diseases [102]. These indicate that *P. gingivalis* may be one of the causes or inducers of liver disease.

Oral infection can activate the host immune system and trigger an inflammatory reaction [107]. When exogenous pathogens enter the oral cavity, they induce the body’s immune system to generate an immune response. If the intrinsic immune response fails to completely remove the pathogen, a subsequent specific adaptive immune response will be triggered to completely remove the pathogen, protect the organism, and form an immune memory that will lead to a faster and stronger immune response when the same pathogen is encountered the next time [108]. If the adaptive immune response also fails to completely clear the invading pathogens, a chronic infectious state will result. Long-term oral infection may lead to continuous activation of the immune system and increase the risk of systemic inflammation. Some possible mechanisms related to liver disease and periodontitis are dysbacteriosis, changes in the level of inflammatory agents, and oxidative stress [109]. The liver of rats with periapical periodontitis, compared with the control group, had higher levels of vitamin C, Na+/K+ATPase, and catalase activity, which indicated that there were oxidative stress reactions and hepatocyte degeneration induced by periapical periodontitis in the liver tissue [110]. In addition, bacteria and their metabolites enter the liver through the portal vein and can interact with the inherent sensors (TLR and NLR) of liver cells and Kupffer cells, resulting in the production of inflammatory mediators and thus causing liver damage.

Modulating gut microbial homeostasis and targeting oral pathogenic bacteria are currently the main therapeutic strategies for liver diseases based on the oral–gut–liver axis. Various therapeutic approaches targeting the microbiota have been developed: antibiotics, probiotics, prebiotics, synthetic probiotics, and fecal microbial transplants, all of which have been recognized as effective treatments for liver and metabolic diseases associated with gut microbiota dysbiosis [111,112]. Dietary fiber has been shown to improve early NAFLD by reducing calorie absorption and correcting imbalances in the gut microbiota [113]. *Lactococcus lactis* treatment modulates the formation, composition, and survival of oral bacterial biofilms, has potent antimicrobial and anti-inflammatory properties, and is also benefical in decreasing LFC, suggesting its promising efficiency in the treatment of NAFLD [114,115].

Also, oral inflammation treatment is one of the important complementary therapies for liver diseases. Kamata et al. conducted a multicenter randomized controlled trial to evaluate the effects of periodontal therapy on patients with NAFLD and discovered that periodontal therapy significantly reduced liver enzyme levels, endotoxin levels, and LFC in patients with NAFLD combined with periodontal disease [85]. A proof-of-concept clinical trial has reported that cirrhotic patients treated with periodontal therapy exhibit improved inflammation and endotoxemia, suggesting that periodontal therapy modulates the gut microbiome in cirrhotic patients and that this alteration appears to provide beneficial effects [62]. More research linking variations in oral microbiota diversity to clinical outcomes and therapeutic responsiveness is expected, and specific oral pathogenic bacteria may serve as biomarkers for gastrointestinal and liver diseases.

## 7. Future Direction

Having a better understanding of the mechanisms of the oral–gut–liver association is an exciting new frontier of research. Microecological dysregulation associated with periodontitis appears to exacerbate hepatic pathology, but the mechanisms of ectopic colonization of oral microbiota in the gut have not been fully elucidated. Further research is required to determine whether oral microbiota can reach the liver via the hematogenous pathway or whether it is indirectly regulated mainly through the oral–gut–liver axis. In addition, periodontitis exhibits intestinal microecological dysregulation, which is similar to the presentation of chronic liver disease [5]. It is still unexplored whether this microecological dysregulation is causally linked to one disease or whether the dysregulation shared by both diseases causes an inflammatory response.

Further prospective studies and sequencing of the entire macrogenomes of the oral and gut microbiomes may be needed in the future to gain a deeper understanding of the taxonomic and functional profiles of the oral and intestinal microbiota. Given the ease of sampling, coupled with the depth of information that can be retrieved from the oral microbiome, such studies may mark a significant shift in our approach to elucidating the role of the microbiome in liver disease [116]. As a result, there will be a greater chance of employing the oral pathogenic bacteria as biomarkers for extra-oral diseases, notably liver diseases, pointing the way to research into the unique functions and mechanisms of the oral–gut–liver axis [7].

## 8. Conclusions

In summary, changes in the abundance of oral pathogenic bacteria may influence the pathogenesis of various chronic liver diseases through the oral–gut–liver axis. Although there is no clinical approach for completely eradicating chronic liver disease, gut microbiota modulation as well as targeting oral pathogenic bacteria are currently the main therapeutic strategies for liver disease based on the oral–gut–liver axis. Some specific oral pathogens may be potential biomarkers for chronic liver disease and have innovative applications in screening, treatment, and prognosis for liver management. This may shed some light on future liver therapeutic strategies; integrating dentistry and clinical medicine will bring important clinical value. Further studies are required to determine the effectiveness and feasibility of the oral–gut–liver axis-based approach discussed in this review in the management of chronic liver disease and to elucidate the depth of the relationship between human physiology and the oral microbiome.

## Figures and Tables

**Table 1:** Characteristics of main oral pathogenic bacteria.

Species	*P. gingivalis*	*S. mutans*	*P. intermedia*	*A. actinomycetemcomitans*	*F. nucleatum*
Phylum	Bacteroidetes	Firmicutes	Bacteroidetes	Proteobacteria	Fusobacteria
Gram stain	Negative	Postive	Postive	Negative	Negative
Respiration characteristic	Anaerobic	Facultative anaerobe	Anaerobic	Facultative anaerobe	Anaerobic
Main location	Gingiva	Plaque	Gingiva	Gingiva	Plaque

**Table 2:** Changes in the oral microbiota related to liver diseases.

Type of Liver Diseases		Microbial Genus of Potential Diagnostic Biomarkers	References	Clinical Significance
ALD		*P. gingivalis*↑	[44]	*P. gingivalis* may be a risk factor for the occurrence/severity of AAH.
		*Fusobacterium↑* *Veillonella↑* *Bifidobacterium↑* *Lactobacillus ↑* *Streptococcus ↑*	[45]	Patients with alcohol-associated cirrhosis and alcoholic hepatitis are enriched for more common pathogenic taxa.
AILD	AIH	*Streptococcus*↑ *Veillonella* ↑ *Leptotrichia*↑	[33]	Oral microbiota-targeted biomarkers may be able to serve as powerful and noninvasive diagnostic tools for AIH.
	AIH	*Veillonella*↑ *Streptococcus*↓	[46]	Further studies of the establishment and modification of the oral microbiota structure may contribute to the development of a therapeutic strategy for patients with AILD.
	PBC	*Eubacterium*↑ *Veillonella*↑ *Fusobacterium*↓		
NAFLD		*P. gingivalis*↑	[47]	*P. gingivalis* induced the activation of hepatic stellate cells (HSCs; key effector cells in liver fibrosis).
		*P. gingivalis* ↑	[44]	*P. gingivalis* can induce the progression of fatty liver disease in HFD-fed mice through the upregulation of the CD36-PPARγ axis.
Cirrhosis		*Bacteroidetes↑* *Spirochaetes↑* *Fusobacteria↑* *Tannerella forsythia↓* *Treponema denticala*↓	[48]	The subgingival plaque microbiome in patients with liver cirrhosis and periodontitis differed from that of otherwise healthy patients with periodontitis.
HCC		*Gammaproteobacteria ↓* *Bacteroidetes↓* *Epsilonproteobacteria↑* *Actinobacteria↑* *Clostridia↑* *Fusobacteria↑*	[31]	The oral microbiome serves as a diagnostic biomarker for HCC.

Upward arrows indicate increasing abundance, downward arrows indicate decreasing abundance.

## Data Availability

Not applicable.

## References

[1] Devarbhavi H., Asrani S.K., Arab J.P., Nartey Y.A., Pose E., Kamath P.S. (2023). Global burden of liver disease: 2023 update. J. Hepatol..

[2] Ma C., Qian A.S., Nguyen N.H., Stukalin I., Congly S.E., Shaheen A.A., Swain M.G., Teriaky A., Asrani S.K., Singh S. (2021). Trends in the Economic Burden of Chronic Liver Diseases and Cirrhosis in the United States: 1996–2016. Am. J. Gastroenterol..

[3] Verma M., Younossi Z. (2021). Integrating Patient-Reported Outcomes within Routine Hepatology Care: A Prompt to Action. Hepatology.

[4] Jones R.M., Neish A.S. (2021). Gut Microbiota in Intestinal and Liver Disease. Annu. Rev. Pathol..

[5] Albuquerque-Souza E., Sahingur S.E. (2022). Periodontitis, chronic liver diseases, and the emerging oral-gut-liver axis. Periodontol. 2000.

[6] Kitamoto S., Nagao-Kitamoto H., Hein R., Schmidt T.M., Kamada N. (2020). The Bacterial Connection between the Oral Cavity and the Gut Diseases. J. Dent. Res..

[7] Imai J., Kitamoto S., Kamada N. (2021). The pathogenic oral-gut-liver axis: New understandings and clinical implications. Expert. Rev. Clin. Immunol..

[8] Tuominen H., Rautava J. (2021). Oral Microbiota and Cancer Development. Pathobiology.

[9] Usui M., Onizuka S., Sato T., Kokabu S., Ariyoshi W., Nakashima K. (2021). Mechanism of alveolar bone destruction in periodontitis—Periodontal bacteria and inflammation. JPN Dent. Sci. Rev..

[10] D’Ercole S., D’Addazio G., Di Lodovico S., Traini T., Di Giulio M., Sinjari B. (2020). *Porphyromonas Gingivalis* Load is Balanced by 0.20% Chlorhexidine Gel. A Randomized, Double-Blind, Controlled, Microbiological and Immunohistochemical Human Study. J. Clin. Med..

[11] Lunar Silva I., Cascales E. (2021). Molecular Strategies Underlying *Porphyromonas gingivalis* Virulence. J. Mol. Biol..

[12] Kwack K.H., Jang E.Y., Yang S.B., Lee J.H., Moon J.H. (2022). Genomic and phenotypic comparison of *Prevotella intermedia* strains possessing different virulence in vivo. Virulence.

[13] Gholizadeh P., Pormohammad A., Eslami H., Shokouhi B., Fakhrzadeh V., Kafil H.S. (2017). Oral pathogenesis of *Aggregatibacter actinomycetemcomitans*. Microb. Pathog..

[14] Lin Y., Gong T., Ma Q., Jing M., Zheng T., Yan J., Chen J., Pan Y., Sun Q., Zhou X. (2022). Nicotinamide could reduce growth and cariogenic virulence of *Streptococcus mutans*. J. Oral. Microbiol..

[15] Tonomura S., Naka S., Tabata K., Hara T., Mori K., Tanaka S., Sumida Y., Kanemasa K., Nomura R., Matsumoto-Nakano M. (2019). Relationship between *Streptococcus mutans* expressing Cnm in the oral cavity and non-alcoholic steatohepatitis: A pilot study. BMJ Open Gastroenterol..

[16] Choi E.Y., Lee J.E., Lee A.R., Choi I.S., Kim S.J. (2022). Nitrooleic acid inhibits macrophage activation induced by lipopolysaccharide from *Prevotella intermedia*. Nutr. Res..

[17] Su Y., Ye L., Hu C., Zhang Y., Liu J., Shao L. (2023). Periodontitis as a promoting factor of T2D: Current evidence and mechanisms. Int. J. Oral. Sci..

[18] Read E., Curtis M.A., Neves J.F. (2021). The role of oral bacteria in inflammatory bowel disease. Nat. Rev. Gastroenterol. Hepatol..

[19] Xiao E., Mattos M., Vieira G.H.A., Chen S., Corrêa J.D., Wu Y., Albiero M.L., Bittinger K., Graves D.T. (2017). Diabetes Enhances, I.L-17 Expression and Alters the Oral Microbiome to Increase Its Pathogenicity. Cell Host Microbe.

[20] Maitre Y., Micheneau P., Delpierre A., Mahalli R., Guerin M., Amador G., Denis F. (2020). Did the Brain and Oral Microbiota Talk to Each Other? A Review of the Literature. J. Clin. Med..

[21] Abe K., Fujita M., Hayashi M., Okai K., Takahashi A., Ohira H. (2020). Gut and oral microbiota in autoimmune liver disease. Fukushima J. Med. Sci..

[22] Graves D.T., Corrêa J.D., Silva T.A. (2019). The Oral Microbiota Is Modified by Systemic Diseases. J. Dent. Res..

[23] Peng X., Cheng L., You Y., Tang C., Ren B., Li Y., Xu X., Zhou X. (2022). Oral microbiota in human systematic diseases. Int. J. Oral. Sci..

[24] Park S.Y., Hwang B.O., Lim M., Ok S.H., Lee S.K., Chun K.S., Park K.K., Hu Y., Chung W.Y., Song N.Y. (2021). Oral-Gut Microbiome Axis in Gastrointestinal Disease and Cancer. Cancers.

[25] Wang J., Feng J., Zhu Y., Li D., Wang J., Chi W. (2022). Diversity and Biogeography of Human Oral Saliva Microbial Communities Revealed by the Earth Microbiome Project. Front. Microbiol..

[26] Mo S., Ru H., Huang M., Cheng L., Mo X., Yan L. (2022). Oral-Intestinal Microbiota in Colorectal Cancer: Inflammation and Immunosuppression. J. Inflamm. Res..

[27] Zhao L., Zhang X., Zhou Y., Fu K., Lau H.C., Chun T.W., Cheung A.H., Coker O.O., Wei H., Wu W.K. (2022). Effect of Entecavir on the Intestinal Microflor. Oncogene.

[28] Proc P., Szczepańska J., Zarzycka B., Szybka M., Borowiec M., Płoszaj T., Fendler W., Chrzanowski J., Zubowska M., Stolarska M. (2021). Evaluation of Changes to the Oral Microbiome Based on 16S rR.NA Sequencing among Children Treated for Cancer. Cancers.

[29] Zhang Y., Niu Q., Fan W., Huang F., He H. (2019). Oral microbiota and gastrointestinal cancer. Onco Targets Ther..

[30] Qi Y., Wu H.M., Yang Z., Zhou Y.F., Jin L., Yang M.F., Wang F.Y. (2022). New Insights into the Role of Oral Microbiota Dysbiosis in the Pathogenesis of Inflammatory Bowel Disease. Dig. Dis. Sci..

[31] Lu Y.X., He C.Z., Wang Y.X., Ai Z.S., Liang P., Yang C.Q. (2021). Effect of Entecavir on the Intestinal Microflora in Patients with Chronic Hepatitis B: A Controlled Cross-Sectional and Longitudinal Real-World Study. Infect. Dis. Ther..

[32] Costa C.F.F.A., Sampaio-Maia B., Araujo R., Nascimento D.S., Ferreira-Gomes J., Pestana M., Azevedo M.J., Alencastre I.S. (2022). Gut Microbiome and Organ Fibrosis. Nutrients.

[33] Rao B., Ren T., Wang X., Wang H., Zou Y., Sun Y., Liu S., Ren Z., Yu Z. (2021). Dysbiosis in the Human Microbiome of Cholangiocarcinoma. Front. Physiol..

[34] Renzulli M., Brandi N., Pecorelli A., Pastore L.V., Granito A., Martinese G., Tovoli F., Simonetti M., Dajti E., Colecchia A. (2022). Segmental Distribution of Hepatocellular Carcinoma in Cirrhotic Livers. Diagn..

[35] Tripathi A., Debelius J., Brenner D.A., Karin M., Loomba R., Schnabl B., Knight R. (2018). The gut-liver axis and the intersection with the microbiome. Nat. Rev. Gastroenterol. Hepatol..

[36] Couto N., Al-Majdoub Z.M., Gibson S., Davies P.J., Achour B., Harwood M.D., Carlson G., Barber J., Rostami-Hodjegan A., Warhurst G. (2020). Quantitative Proteomics of Clinically Relevant Drug-Metabolizing Enzymes and Drug Transporters and Their Intercorrelations in the Human Small Intestine. Drug Metab. Dispos..

[37] Yamazaki K., Kato T., Tsuboi Y., Miyauchi E., Suda W., Sato K., Nakajima M., Yokoji-Takeuchi M., Yamada-Hara M., Tsuzuno T. (2021). Oral Pathobiont-Induced Changes in Gut Microbiota Aggravate the Pathology of Nonalcoholic Fatty Liver Disease in Mice. Front. Immunol..

[38] Tilg H., Cani P.D., Mayer E.A. (2016). Gut microbiome and liver diseases. Gut.

[39] Brignardello J., Morales P., Diaz E., Romero J., Brunser O., Gotteland M. (2010). Pilot study: Alterations of intestinal microbiota in obese humans are not associated with colonic inflammation or disturbances of barrier function. Aliment. Pharmacol. Ther..

[40] Farhadi A., Gundlapalli S., Shaikh M., Frantzides C., Harrell L., Kwasny M.M., Keshavarzian A. (2008). Susceptibility to gut leakiness: A possible mechanism for endotoxaemia in non-alcoholic steatohepatitis. Liver Int..

[41] Acharya C., Sahingur S.E., Bajaj J.S. (2017). Microbiota, cirrhosis, and the emerging oral-gut-liver axis. J.CI Insight.

[42] Qin N., Yang F., Li A., Prifti E., Chen Y., Shao L., Guo J., Le Chatelier E., Yao J., Wu L. (2014). Alterations of the human gut microbiome in liver cirrhosis. Nature.

[43] Kakiyama G., Hylemon P.B., Zhou H., Pandak W.M., Heuman D.M., Kang D.J., Takei H., Nittono H., Ridlon J.M., Fuchs M. (2014). Colonic inflammation and secondary bile acids in alcoholic cirrhosis. Am. J. Physiol. Gastrointest. Liver Physiol..

[44] Ahn J.S., Yang J.W., Oh S.J., Shin Y.Y., Kang M.J., Park H.R., Seo Y., Kim H.S. (2021). *Porphyromonas gingivalis* exacerbates the progression of fatty liver disease via C.D36-P.PA.Rγ pathway. BMB Rep..

[45] Fairfield B., Schnabl B. (2020). Gut dysbiosis as a driver in alcohol-induced liver injury. JHEP Rep..

[46] Abe K., Takahashi A., Fujita M., Imaizumi H., Hayashi M., Okai K., Ohira H. (2018). Dysbiosis of oral microbiota and its association with salivary immunological biomarkers in autoimmune liver disease. PLoS ONE.

[47] Nagasaki A., Sakamoto S., Chea C., Ishida E., Furusho H., Fujii M., Takata T., Miyauchi M. (2020). Odontogenic infection by *Porphyromonas gingivalis* exacerbates fibrosis in NASH via hepatic stellate cell activation. Sci. Rep..

[48] Jensen A., Ladegaard Grønkjær L., Holmstrup P., Vilstrup H., Kilian M. (2018). Unique subgingival microbiota associated with periodontitis in cirrhosis patients. Sci. Rep..

[49] European Association for the Study of the Liver (2018). EASL Clinical Practice Guidelines: Management of alcohol-related liver disease. J. Hepatol..

[50] Roerecke M., Shield K.D., Higuchi S., Yoshimura A., Larsen E., Rehm M.X., Rehm J. (2015). Estimates of alcohol-related oesophageal cancer burden in Japan: Systematic review and meta-analyses. Bull. World Health Organ..

[51] Li F., McClain C.J., Feng W. (2019). Microbiome dysbiosis and alcoholic liver disease. Liver Res..

[52] Fan X., Peters B.A., Jacobs E.J., Gapstur S.M., Purdue M.P., Freedman N.D., Alekseyenko A.V., Wu J., Yang L., Pei Z. (2018). Drinking alcohol is associated with variation in the human oral microbiome in a large study of American adults. Microbiome.

[53] Zhou Y., Vatsalya V., Gobejishvili L., Lamont R.J., McClain C.J., Feng W. (2018). *Porphyromonas gingivalis* as a Possible Risk Factor in the Development/Severity of Acute Alcoholic Hepatitis. Hepatol. Commun..

[54] Sarin S.K., Pande A., Schnabl B. (2019). Microbiome as a therapeutic target in alcohol-related liver disease. J. Hepatol..

[55] Kuraji R., Shiba T., Dong T.S., Numabe Y., Kapila Y.L. (2023). Periodontal treatment and microbiome-targeted therapy in management of periodonti-tis-related nonalcoholic fatty liver disease with oral and gut dysbiosis. World J. Gastroenterol..

[56] Kamata Y., Kessoku T., Shimizu T., Kobayashi T., Kurihashi T., Sato S., Kuraji S., Aoyama N., Iwasaki T., Takashiba S. (2020). Efficacy and safety of PERIOdontal treatment versus usual care for Nonalcoholic liver disease: Protocol of the PERION multicenter, two-arm, open-label, randomized trial. Trials.

[57] Yoneda M., Naka S., Nakano K., Wada K., Endo H., Mawatari H., Imajo K., Nomura R., Hokamura K., Ono M. (2012). Involvement of a periodontal pathogen, *Porphyromonas gingivalis* on the pathogenesis of non-alcoholic fatty liver disease. BMC Gastroenterol..

[58] Nagasaki A., Sakamoto S., Arai T., Kato M., Ishida E., Furusho H., Fujii M., Takata T., Miyauchi M. (2021). Elimination of *Porphyromonas gingivalis* inhibits liver fibrosis and inflammation in NASH. J. Clin. Periodontol..

[59] Yao C., Lan D., Li X., Wang Y., Qi S., Liu Y. (2023). *Porphyromonas gingivalis* is a risk factor for the development of nonalcoholic fatty liver disease via ferroptosis. Microbes Infect..

[60] Seyama M., Yoshida K., Yoshida K., Fujiwara N., Ono K., Eguchi T., Kawai H., Guo J., Weng Y., Haoze Y. (2020). Outer membrane vesicles of *Porphyromonas gingivalis* attenuate insulin sensitivity by delivering gingipains to the liver. Biochim. Biophys. Acta Mol. Basis Dis..

[61] Seen S. (2021). Chronic liver disease and oxidative stress—A narrative review. Expert. Rev. Gastroenterol. Hepatol..

[62] Bajaj J.S., Matin P., White M.B., Fagan A., Deeb J.G., Acharya C., Dalmet S.S., Sikaroodi M., Gillevet P.M., Sahingur S.E. (2018). Periodontal therapy favorably modulates the oral-gut-hepatic axis in cirrhosis. Am. J. Physiol. Gastrointest. Liver Physiol..

[63] Costa F.O., Lages E.J.P., Lages E.M.B., Cota L.O.M. (2019). Periodontitis in individuals with liver cirrhosis: A case-control study. J. Clin. Periodontol..

[64] Ladegaard Grønkjær L., Holmstrup P., Schou S., Jepsen P., Vilstrup H. (2018). Severe periodontitis and higher cirrhosis mortality. United Eur. Gastroenterol. J..

[65] Di Profio B., Villar C.C., Saraiva L., Ortega K.L., Pannuti C.M. (2017). Is periodontitis a risk factor for infections in cirrhotic patients?. Med. Hypotheses.

[66] Omura Y., Kitamoto M., Hyogo H., Yamanoue T., Tada Y., Boku N., Nishisaka T., Miyauchi M., Takata T., Chayama K. (2016). Morbidly obese patient with non-alcoholic steatohepatitis-related cirrhosis who died from sepsis caused by dental infection of *Porphyromonas gingivalis*: A case report. Hepatol. Res..

[67] Hernandez B.Y., Zhu X., Risch H.A., Lu L., Ma X., Irwin M.L., Lim J.K., Taddei T.H., Pawlish K.S., Stroup A.M. (2022). Oral Cyanobacteria and Hepatocellular Carcinoma. Cancer Epidemiol. Biomark. Prev..

[68] Thistle J.E., Yang B., Petrick J.L., Fan J.H., Qiao Y.L., Abnet C.C., Taylor P.R., McGlynn K.A. (2018). Association of tooth loss with liver cancer incidence and chronic liver disease mortality in a rural Chinese population. PLoS ONE.

[69] Yang B., Petrick J.L., Abnet C.C., Graubard B.I., Murphy G., Weinstein S.J., Männistö S., Albanes D., McGlynn K.A. (2017). Tooth loss and liver cancer incidence in a Finnish cohort. Cancer Causes Control..

[70] Schwabe R.F., Greten T.F. (2020). Gut microbiome in H.CC—Mechanisms, diagnosis and therapy. J. Hepatol..

[71] Hedgpeth D.C., Zhang X., Jin J., Leite R.S., Krayer J.W., Huang Y. (2015). Periodontal, C.D14 mR.NA expression is downregulated in patients with chronic periodontitis and type 2 diabetes. BMC Oral. Health.

[72] Wu J.S., Zheng M., Zhang M., Pang X., Li L., Wang S.S., Yang X., Wu J.B., Tang Y.J., Tang Y.L. (2018). *Porphyromonas gingivalis* Promotes 4-Nitroquinoline-1-Oxide-Induced Oral Carcinogenesis with an Alteration of Fatty Acid Metabolism. Front. Microbiol..

[73] Lin Y.W., Li C.F., Chen H.Y., Yen C.Y., Lin L.C., Huang C.C., Huang H.Y., Wu P.C., Chen C.H., Chen S.C. (2012). The expression and prognostic significance of hepatoma-derived growth factor in oral cancer. Oral. Oncol..

[74] Rao B.C., Lou J.M., Wang W.J., Li A., Cui G.Y., Yu Z.J., Ren Z.G. (2020). Human microbiome is a diagnostic biomarker in hepatocellular carcinoma. Hepatobiliary Pancreat. Dis. Int..

[75] du Teil Espina M., Gabarrini G., Harmsen H.J.M., Westra J., van Winkelhoff A.J., van Dijl J.M. (2019). Talk to your gut: The oral-gut microbiome axis and its immunomodulatory role in the etiology of rheumatoid arthritis. FEMS Microbiol. Rev..

[76] Tanaka A., Leung P.S.C., Gershwin M.E. (2019). Pathogen infections and primary biliary cholangitis. Clin. Exp. Immunol..

[77] Shimoda S., Nakamura M., Ishibashi H., Hayashida K., Niho Y.H.L.A. (1995). D.RB4 0101-restricted immunodominant T cell autoepitope of pyruvate dehydrogenase complex in primary biliary cirrhosis: Evidence of molecular mimicry in human autoimmune diseases. J. Exp. Med..

[78] Rao B., Lou J., Lu H., Liang H., Li J., Zhou H., Fan Y., Zhang H., Sun Y., Zou Y. (2021). Oral Microbiome Characteristics in Patients With Autoimmune Hepatitis. Front. Cell Infect. Microbiol..

[79] Han Y.W. (2015). *Fusobacterium nucleatum*: A commensal-turned pathogen. Curr. Opin. Microbiol..

[80] Durand R., Roufegarinejad A., Chandad F., Rompré P.H., Voyer R., Michalowicz B.S., Emami E. (2019). Dental caries are positively associated with periodontal disease severity. Clin. Oral. Investig..

[81] Kilian M. (2018). The oral microbiome—Friend or foe?. Eur. J. Oral. Sci..

[82] Sälzer S., Graetz C., Dörfer C.E., Slot D.E., Van der Weijden F.A. (2020). Contemporary practices for mechanical oral hygiene to prevent periodontal disease. Periodontol. 2000.

[83] Chapple I.L., Van der Weijden F., Doerfer C., Herrera D., Shapira L., Polak D., Madianos P., Louropoulou A., Machtei E., Donos N. (2015). Primary prevention of periodontitis: Managing gingivitis. J. Clin. Periodontol..

[84] Kim J.Y., Park Y.M., Lee G.N., Song H.C., Ahn Y.B., Han K., Ko S.H. (2021). Association between toothbrushing and non-alcoholic fatty liver disease. PLoS ONE.

[85] Kamata Y., Kessoku T., Shimizu T., Sato S., Kobayashi T., Kurihashi T., Morozumi T., Iwasaki T., Takashiba S., Hatanaka K. (2022). Periodontal Treatment and Usual Care for Nonalcoholic Fatty Liver Disease: A Multicenter, Randomized Controlled Trial. Clin. Transl. Gastroenterol..

[86] Niu J.Y., Yin I.X., Mei M.L., Wu W.K.K., Li Q.L., Chu C.H. (2021). The multifaceted roles of antimicrobial peptides in oral diseases. Mol. Oral. Microbiol..

[87] Signat B., Roques C., Poulet P., Duffaut D. (2011). Fusobacterium nucleatum in periodontal health and disease. Curr. Issues Mol. Biol..

[88] Tonelli A., Lumngwena E.N., Ntusi N.A.B. (2023). The oral microbiome in the pathophysiology of cardiovascular disease. Nat. Rev. Cardiol..

[89] Pütsep K., Carlsson G., Boman H.G., Andersson M. (2002). Deficiency of antibacterial peptides in patients with morbus Kostmann: An observation study. Lancet.

[90] Gäbele E., Dostert K., Hofmann C., Wiest R., Schölmerich J., Hellerbrand C., Obermeier F. (2011). DSS induced colitis increases portal LPS levels and enhances hepatic inflammation and fibrogenesis in ex-perimental NASH. J. Hepatol..

[91] Wang Q., Kim S.Y., Matsushita H.W., Wang Z., Pandyarajan V., Matsuda M., Ohashi K., Tsuchiya T., Roh Y.S., Kiani C. (2021). Oral administration of P.EGylated, T.LR7 ligand ameliorates alcohol-associated liver disease via the induction of I.L-22. Proc. Natl. Acad. Sci. USA.

[92] Ding X., Bian D., Li W., Xie Y., Li X., Lv J., Tang R. (2021). Host defense peptide L.L-37 is involved in the regulation of cell proliferation and production of pro-inflammatory cytokines in hepatocellular carcinoma cells. Amino Acids.

[93] Sanders M.E., Merenstein D.J., Reid G., Gibson G.R., Rastall R.A. (2019). Probiotics and prebiotics in intestinal health and disease: From biology to the clinic. Nat. Rev. Gastroenterol. Hepatol..

[94] Maitre Y., Mahalli R., Micheneau P., Delpierre A., Guerin M., Amador G., Denis F. (2021). Pre and Probiotics Involved in the Modulation of Oral Bacterial Species: New Therapeutic Leads in Mental Disorders?. Microorganisms.

[95] Bashiardes S., Shapiro H., Rozin S., Shibolet O., Elinav E. (2016). Non-alcoholic fatty liver and the gut microbiota. Mol. Metab..

[96] Aron-Wisnewsky J., Warmbrunn M.V., Nieuwdorp M., Clément K. (2020). Nonalcoholic Fatty Liver Disease: Modulating Gut Microbiota to Improve Severity?. Gastroenterology.

[97] Matsubara V.H., Bandara H.M., Ishikawa K.H., Mayer M.P., Samaranayake L.P. (2016). The role of probiotic bacteria in managing periodontal disease: A systematic review. Expert. Rev. Anti. Infect. Ther..

[98] Sharpton S.R., Maraj B., Harding-Theobald E., Vittinghoff E., Terrault N.A. (2019). Gut microbiome-targeted therapies in nonalcoholic fatty liver disease: A systematic review, meta-analysis, and meta-regression. Am. J. Clin. Nutr..

[99] Ahn S.B., Jun D.W., Kang B.K., Lim J.H., Lim S., Chung M.J. (2019). Randomized, Double-blind, Placebo-controlled Study of a Multispecies Probiotic Mixture in Nonalcoholic Fatty Liver Disease. Sci. Rep..

[100] Zhao X., Liu J., Zhang C., Yu N., Lu Z., Zhang S., Li Y., Li Q., Liu J., Liu D. (2021). *Porphyromonas gingivalis* exacerbates ulcerative colitis via *Porphyromonas gingivalis* peptidylarginine deiminase. Int. J. Oral. Sci..

[101] Bregaint S., Boyer E., Fong S.B., Meuric V., Bonnaure-Mallet M., Jolivet-Gougeon A. (2022). *Porphyromonas gingivalis* outside the oral cavity. Odontology.

[102] Ding L.Y., Liang L.Z., Zhao Y.X., Yang Y.N., Liu F., Ding Q.R., Luo L.J. (2019). *Porphyromonas gingivalis*-derived lipopolysaccharide causes excessive hepatic lipid accumulation via activating N.F-κB and J.NK signaling pathways. Oral. Dis..

[103] Olsen I., Yilmaz Ö. (2019). Possible role of *Porphyromonas gingivalis* in orodigestive cancers. J. Oral. Microbiol..

[104] Liu Y., Huang W., Dai K., Liu N., Wang J., Lu X., Ma J., Zhang M., Xu M., Long X. (2022). Inflammatory response of gut, spleen, and liver in mice induced by orally administered *Porphyromonas gingivalis*. J. Oral. Microbiol..

[105] Mohammed H., Varoni E.M., Cochis A., Cordaro M., Gallenzi P., Patini R., Staderini E., Lajolo C., Rimondini L., Rocchetti V. (2018). Oral Dysbiosis in Pancreatic Cancer and Liver Cirrhosis: A Review of the Literature. Biomedicines..

[106] Ohyama H., Nakasho K., Yamanegi K., Noiri Y., Kuhara A., Kato-Kogoe N., Yamada N., Hata M., Nishimura F., Ebisu S. (2009). An unusual autopsy case of pyogenic liver abscess caused by periodontal bacteria. Jpn. J. Infect. Dis..

[107] Brock M., Bahammam S., Sima C. (2022). The Relationships Among Periodontitis, Pneumonia and COVID-19. Front. Oral. Health.

[108] Pone E.J., Zan H., Zhang J., Al-Qahtani A., Xu Z., Casali P. (2010). Toll-like receptors and B-cell receptors synergize to induce immunoglobulin class-switch D.NA recombination: Relevance to microbial antibody responses. Crit. Rev. Immunol..

[109] Han P., Sun D., Yang J. (2016). Interaction between periodontitis and liver diseases. Biomed. Rep..

[110] Barcelos R.C.S., Rosa H.Z., Roversi K., Tibúrcio-Machado C.D.S., Inchaki P.T., Burger M.E., Bier C.A.S. (2020). Apical periodontitis induces changes on oxidative stress parameters and increases Na+/K+-A.TPase activity in adult rats. Arch. Oral. Biol..

[111] Chen H.T., Huang H.L., Li Y.Q., Xu H.M., Zhou Y.J. (2020). Therapeutic advances in non-alcoholic fatty liver disease: A microbiota-centered view. World J. Gastroenterol..

[112] Boursier J., Mueller O., Barret M., Machado M., Fizanne L., Araujo-Perez F., Guy C.D., Seed P.C., Rawls J.F., David L.A. (2016). The severity of nonalcoholic fatty liver disease is associated with gut dysbiosis and shift in the metabolic function of the gut microbiota. Hepatology.

[113] Baker J.L., Mark Welch J.L., Kauffman K.M., McLean J.S., He X. (2023). The oral microbiome: Diversity, biogeography and human health. Nat. Rev. Microbiol..

[114] Sookoian S., Salatino A., Castaño G.O., Landa M.S., Fijalkowky C., Garaycoechea M., Pirola C.J. (2020). Intrahepatic bacterial metataxonomic signature in non-alcoholic fatty liver disease. Gut.

[115] Van Pijkeren J.P., Neoh K.M., Sirias D., Findley A.S., Britton R.A. (2012). Exploring optimization parameters to increase ssD.NA recombineering in *Lactococcus lactis* and *Lactobacillus reuteri*. Bioengineered.

[116] Tawfik S.A., Azab M., Ramadan M., Shabayek S., Abdellah A., Al Thagfan S.S., Salah M. (2023). The Eradication of *Helicobacter pylori* Was Significantly Associated with Compositional Patterns of Orointestinal Axis Microbiota. Pathogens.

